# Unraveling Oral Dysbiosis: Microbial Complexity in Common Oral Diseases

**DOI:** 10.1002/mbo3.70305

**Published:** 2026-05-24

**Authors:** Zixi Kang, Hong Huang, Jin Lin, Yanan Niu, Jiaxin Chen, Jiaxuan Tang, Zhengyuan Hu, Peng Liu, Jun Qu

**Affiliations:** ^1^ Institute of Pathogenic Biology, Basic Medical School, Hengyang Medical School University of South China Hengyang China; ^2^ Affiliated Hengyang Hospital of Hunan Normal University & Hengyang Central Hospital Hengyang Hunan China

**Keywords:** clinical implications, dysbiosis, microbiome, oral diseases, pathogenesis

## Abstract

The oral microbiome is highly intricate, hosting billions of bacteria and other microorganisms that form biofilms on various oral surfaces. An imbalanced ecological relationship between the microbial community and the host can lead to various oral diseases. This narrative review explores the current understanding of the correlation between the microbiome and oral diseases. The main body of this manuscript is divided into seven parts, including a review of current research on oral microbial colonization and early life development, an introduction to five common oral diseases related to microorganisms, and a discussion on the relationship between dental caries and periodontal disease at the microbial level. Our aim in presenting this review is to offer a valuable resource for further research on the role of oral microorganisms in diagnosing and treating oral diseases. The oral microbiome's significant impact and diversity characteristics on health and disease have been recognized; however, there remains a severe lack of systematic understanding of its functions, host interactions, and environmental factors. Comprehensive research is urgently needed to elucidate the mechanisms that maintain its ecological balance, providing a scientific foundation for the precise prevention and control of oral diseases. This review comprehensively synthesizes current knowledge regarding oral microbial dysbiosis in the context of the major oral diseases mentioned and proposes a conceptual framework grounded in microbial ecology to elucidate disease progression and guide therapeutic strategies.

## Graphical abstract

The oral microbiome undergoes dynamic establishment from birth, maturing into a resilient ecosystem. Perturbation of this homeostasis—driven by dietary, immunological, and anatomical factors—triggers dysbiosis, which underpins the pathogenesis of major oral diseases, including dental caries, periodontal disease, salivary gland disorders, oral mucosal lesions, and oral cancer. This review synthesizes microbial involvement across these conditions within a unified ecological perspective, aiming to inform future microbiota‐targeted preventive and therapeutic strategies.

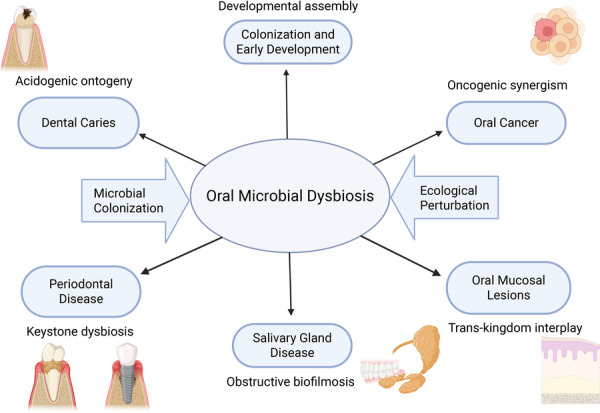

## 1 Introduction

Improved living standards and diets have paradoxically led to worsening oral health, highlighting the need for cost‐effective preventive measures (de Abreu et al. 2021). There are more than 770 characteristic species of oral microorganisms, second only to the intestinal mucosa. The oral microbiota is one of the most diverse groups in the human body, thanks to the numerous characteristics of the oral environment, which encompass surface diversity (e.g., mucosal surfaces, hard tissues, and salivary glands), wettability, and unique physicochemical properties (Pedersen et al. 2018; Tuominen and Rautava 2021). The impact of bacteria on oral diseases has long been recognized (Kunath et al. 2024; A. Gupta et al. 2024). The interaction between the microbial community and the human host plays a crucial role in maintaining the balance of the oral microenvironment. It regulates immune responses, modifies food intake, participates in vitamin biosynthesis, protects against exogenous pathogens, and produces antimicrobial substances. Disruptions in homeostasis and changes in bacterial composition, influenced by internal and external factors, can contribute to the development of oral diseases such as caries, periodontal disease, and oral cancer (S.‐C. Luo et al. 2024; Escobar‐Arregocés et al. 2024; S. Wang, Tan, et al. 2024); however, a systematic understanding of how these shifts originate and propagate remains elusive. In this review, we introduce a conceptual framework grounded in microbial ecology to synthesize current findings across five major oral diseases. Shifting the focus from individual pathogens, we emphasize community‐level dysbiosis, the ecological drivers that orchestrate this disruption (e.g., diet, immunity, and anatomy), and its resultant functional consequences (e.g., acid production, inflammation, and carcinogen generation). This framework aims to uncover unifying ecological principles that govern oral disease pathogenesis and to inform the development of future microbiota‐targeted preventive and therapeutic strategies. Currently, there is a substantial body of research on the relationship between the oral microbiome and diseases, and an urgent need exists for in‐depth, integrated analyses of specific, important diseases. Although the earliest relevant references can be traced back to 1950, considering PubMed search results from the last decade, this paper focuses on five common oral diseases related to microorganisms: dental caries, periodontal disease, salivary gland disease, oral mucosal disease, and oral cancer. It reviews the development of oral microbiota colonization in early human life and its relationship with these diseases, including the main pathogenic microorganisms, pathogenesis, and other related factors of each disease. The five oral diseases examined in this review represent major public health burdens globally, underscoring the need for a comprehensive synthesis of their microbial etiology (Petersen et al. 2005; Pan et al. 2022; Rajasekaran et al. 2024). This review comprehensively summarizes these diseases from a microbiological perspective, briefly outlining first‐line therapies, commonly used prevention strategies, and several emerging treatment strategies. It aims to provide a theoretical reference for developing new clinical therapies and preventive measures by integrating the latest understanding in microbiology. However, it should be noted that most current studies still primarily identify the correlation between microbial communities and disease, and their potential causal mechanisms and viability as clinical targets require further validation through in‐depth functional experiments and rigorous clinical trials.

## 2 Origin of the Normal Oral Microbiota: Colonization and Early Development

A healthy oral environment can be seen as a balanced microbial community. To better understand the relationship between microbiota and disease, it is crucial to first understand the microorganisms present in a healthy oral environment. Obviously, with advancing age, the routes of contact between the human body and various microorganisms become increasingly abundant. Broadly, to minimize the impact of the external environment, one should pay attention to the microorganisms in the mouth, particularly during the colonization period of microorganisms in newborns.

The origin of the newborn's oral microbiota is debated. While microbes have been found in meconium, challenging the “sterile womb” hypothesis, subsequent studies suggest the placental microbiome may result from contamination (Perez‐Muñoz et al. 2017; Bushman 2019; Gschwind et al. 2020; Sterpu et al. 2021). Therefore, strict aseptic sampling research is necessary to identify the placental microbiome. Nevertheless, one cannot deny the connection between oral microorganisms and the placenta: Aagaard et al. (2014), who examined more than 300 placental biopsy samples, revealed in their study that the microbiome present in the placenta closely resembled that found in the tongue and tonsils. This finding suggests a potential role of the placenta in providing microorganisms to the newborn's mouth. Analysis of meconium bacterial composition has shown that the newborn's microbiota is acquired postnatally and not during intrauterine development. The placenta's role in this process has been elucidated by Kaan et al. (2021), who proposed that maternal oral microorganisms could reach the placenta via the circulatory system, where they are captured by immune cells and subsequently stimulate the fetal immune system to develop tolerance toward these microorganisms. Notably, this mechanism prevents the fetus from mounting a strong immune response to these microorganisms upon colonization after birth. Hence, a mother's oral microbiota plays a crucial role in determining the oral health of her fetus.

The oral cavity represents a complex ecosystem housing diverse microbial communities across various ecological niches. During the edentulous stage, the oral flora predominantly colonizes the mucosal surfaces, with the greatest abundance of *Firmicutes* in saliva observed during this period. As primary teeth erupt, there is a significant increase in oral colonization sites associated with hard tissues, leading to a rise in both abundance and diversity of the oral microbiome. The prevalence of *Fusobacteria*, *Firmicutes, Proteobacteria,* and other heterotrophs gradually escalates. The eruption of teeth correlates with an increase in the alpha diversity of the microbial community in saliva, resulting in compositional changes. Genera such as *Fusobacterium*, *Neisseria*, *Lautropia*, *Actinomyces*, and *Corynebacterium* exhibit heightened abundance. As the similarity between oral microbial communities in children and adults grows stronger, the oral microbiome matures from an embryonic form to a more resilient state, demonstrating a certain level of resistance to foreign microbial invasions (J. Xiao et al. 2020).

**Figure 1:**
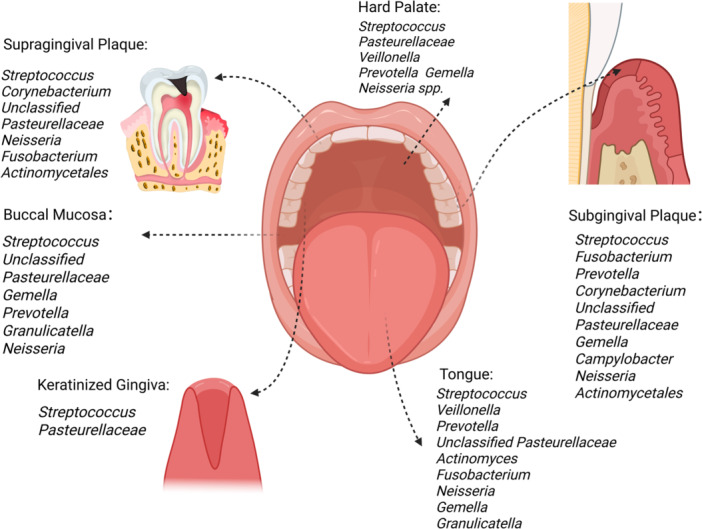
Different microbiota at oral ecological loci. This schematic image shows the microbial species distributed in six different oral anatomical sites, with each arrow connecting the oral cavity with its corresponding anatomical site. It intuitively displays the location of each anatomical site in the oral cavity, while common microorganisms are displayed on one side of each anatomical site. This figure was created in BioRender.

Prior to the eruption of the second molars, the overall oral microbiota is already very complex, consisting of five major phyla (*Firmicutes*, *Fusobacteria*, *Proteobacteria*, *Bacteroidetes*, and *Actinobacteria*) and seven genera (*Leptotrichia, Streptococcus, Actinobacteria, Prevotella, Porphyromonas, Neisseria*, and *Veillonella*), and is unaffected by the presence or absence of abnormal microbial colonization such as dental caries (Xu et al. 2014). By 3 years, the oral microbiome includes six bacterial phyla: *Firmicutes, Proteobacteria, Actinobacteria, Bacteroidetes, Fusobacteria,* and *Spirochaetes*, with a particularly high prevalence of *Proteobacteria*—in particular those of the *Gammaproteobacteria* class (i.e., of the *Pseudomonaceae, Moraxellaceae, Pasteurellaceae,* and *Enterobacteriaceae* families). When adulthood is reached, the types of microorganisms in the oral cavity become even more complex, and the bacterial species on the surface of the oral mucosa vary depending on the anatomical location (Figure 1); for example, *Streptococcus* and *Corynebacterium* are distributed on the supragingival plaque, along with unclassified *Pasteurellaceae* and other microorganisms. In addition, *Streptococcus*, *Fusobacterium*, and *Prevotella*, unclassified *Pasteurellaceae*, *Gemella*, *Campylobacter*, *Neisseria*, *Actinomycota*, and *Corynebacterium* are distributed on the subgingival plaque, while microorganisms such as *Streptococcus* and *Pasteurellaceae* can be found on the hard palate and those such as *Gemella* and *Prevotella* are distributed on the cheek mucosa. Finally, *Streptococcus* and *Pasteurellaceae* may be found on keratinized gingiva, while *Streptococcus*, *Veillonella*, and other such microorganisms are identifiable on both the hard palate and tongue (Tuominen and Rautava 2021).

Caufield et al. (1993) discussed the concept of a “window of infectivity” in their study on how children acquire *Mutans streptococci*. They believe that *M. streptococci* do not immediately colonize the oral cavity after birth but instead are more likely to spread and successfully establish themselves during a specific period. This critical window typically occurs between 19 and 31 months of age in children, at an average of around 26 months, which coincides with the peak of first primary molar eruption. Successfully blocking cariogenic bacteria before or during this window could significantly reduce the risk of future dental caries in children. This insight may inspire future strategies for preventing dental caries in children.

## 3 Dental Caries

Dental caries is one of the most common complex noncommunicable diseases affecting the oral cavity (Clarkson et al. 2021). It is characterized by the demineralization of inorganic substances in teeth and the decomposition of organic substances through a multifactor process mainly driven by bacteria. This process leads to chronic and progressive degradation of the tooth's hard tissue. As a multifactorial ailment, it is influenced by a range of factors such as microbial, genetic, immunological, behavioral, and environmental elements that converge to instigate and progress the onset of dental caries (Aas et al. 2008; Selwitz et al. 2007). The prevailing theory regarding caries etiology is the four primary factors theory, which posits that bacteria, the oral environment, host factors, and time collectively interact, with bacteria serving as the initiating factor and their presence a necessary condition for caries development. The specific microorganisms closely linked to dental caries vary among different age groups. Next, the types of cariogenic microorganisms in children, adults, and the elderly will be briefly discussed.

Early childhood caries is a prevalent chronic childhood disease worldwide (Anderson 2002). It not only causes severe pain but also harms the health‐related quality of life of children around the world (Hallett and O'Rourke 2006; Alm et al. 2007; X. Wang, Ghanbarzadegan, et al. 2024; Y. Liu et al. 2024; Orhan et al. 2024). In a study conducted by Sun et al. (2018), no specific “caries‐specific” bacteria were identified; however, there were notable differences in the abundance of “caries‐associated” bacterial species between children with caries and those without. Of note, *Bacteroidales* and *Prevotella* were found to be highly prevalent in the caries group, suggesting their potential role as caries‐causing microorganisms. Conversely, *Flavobacteriaceae*, *Bergeyella*, and *Corynebacterium* were more abundant in the healthy group, indicating their association with oral health (Sun et al. 2018). A. Luo et al. (2012) used Human Oral Microbe Identification Microarray to analyze saliva, finding higher levels of *Phytophthora*, *Clostridium carbonicum*, *Campylobacter*, and other genera in caries‐active children. Similarly, in a study by Ma et al. (2015), *Streptococcus mutans*, *Porphyromonas, Actinomyces*, and *Bacillus mucilaginous* were more frequently detected in the oral cavities of children with early childhood caries.

Scholars refer to the phenomenon where bacteria can survive in challenging environments but cannot grow on standard culture media as the state of being able to grow but not be cultured (Pazos‐Rojas et al. 2023). Many bacterial species can enter this state under pressure (Pazos‐Rojas et al. 2023). This may serve as a reference for a more comprehensive understanding of oral microbiota, suggesting that some microorganisms that have not yet been isolated and cultured remain to be discovered; there is also the possibility of asymptomatic carrying of microorganisms. If we only focus on noncultivable techniques, then recent research on *Scardovia wiggsiae* can serve as an example. In the past decade, microorganisms related to early childhood dental caries have become a research hotspot. Chandna et al. (2018) isolated *S. wiggsiae* from the saliva of children with early dental caries using real‐time reverse transcriptase polymerase chain reaction, which can serve as a reference for the development of new antimicrobial drugs targeting this organism and provide new ideas for the prevention and treatment of dental caries in children. As a representative of the new frontier of microbial etiology in early childhood caries, many scholars have proposed combining the detection of other specific microorganisms related to early childhood caries, such as *Bifidobacterium* and *S. mutans*, to accurately predict the risk of childhood caries (Vacharaksa et al. 2015; Tantikalchan and Mitrakul 2022).

Also of interest, Aas et al. (2008) conducted a study examining the microbial composition of healthy and carious states in young permanent teeth. Using 16S ribosomal RNA (rRNA) gene library analysis, they compared the microbial profiles of healthy enamel surfaces, white lesion surface plaques, cavitary dentin, and deep dentin plaques in individuals with and without caries. Their study revealed the prevalent presence of *S. mutans* in dentin and deep dentin plaques of cavitated lesions (Aas et al. 2008). Additionally, these authors identified strains of *Veyronella*, *Lactobacillus*, *Bifidobacterium*, *Propionibacterium*, low‐pH nonmutant *Streptococcus*, and *Actinomycetes* as potentially significant contributors to caries development (Aas et al. 2008). A separate study on bacterial diversity and the community structure of supragingival plaques in adults with varying dental health statuses highlighted distinct bacterial profiles. Notably, health‐related bacteria such as *Corynebacterium, Cardiobacterium, Fusobacterium, Leptotrichia, Lachnoanaerobaculum*, and *Aggregatibacter* were prevalent in individuals with good dental health, while caries‐associated bacteria, including *Veillonella, Prevotella, Campylobacter, Eikenella, Catonella, Fretibacterium*, and *Dialister*, among others, were significantly more abundant in individuals with caries (C. Xiao, Ran, et al. 2016).

Elderly individuals often experience gum recession and exposed root surfaces, increasing their susceptibility to root caries. Preza et al. (2008) highlighted the complexity of the microflora associated with root surface caries, surpassing previous assumptions. They identified not only *S. mutans*, *Lactobacilli*, and *Actinomyces* spp.—commonly linked to dental caries—but also suggested the potential involvement of *Atobacter, Olsenia, Pseudomonas lactis, Propionibacterium*, and *Selenomonas* in the development of root surface caries (Preza et al. 2008). Conversely, in a study by L. Chen et al. (2015), *Propionibacterium acidifaciens, S. mutans, Olsenella profusa, Prevotella multisaccharivorax*, and *Lactobacillus crispatus* were identified as core microorganisms associated with root surface caries.

Certain cariogenic microorganisms, such as *S. mutans*, *Lactobacilli*, *Actinomyces* spp., and *Prevotella*, are found consistently across all age groups affected by caries. However, the specific microorganisms closely linked to dental caries vary among age groups—for instance, *Propionibacterium* and *Olsenella* appear predominantly detected in root caries among the elderly.

Diet, especially sucrose intake, significantly influences the formation of cariogenic biofilms and dental plaque (Malin et al. 2024; van Loveren and Duggal 2001; Gao et al. 2024; Liang et al. 2024). Alterations in the oral environment, such as frequent exposure to high sugar concentrations, decrease plaque pH due to the presence of acidophilic and acidogenic microorganisms. In an acidic environment, cariogenic microorganisms thrive and dominate the biofilm, utilizing fermentable carbohydrates to generate acids, thereby lowering the local pH within the biofilm. Concurrently, the acid‐neutralizing capacity of saliva is diminished. This sustained acidic environment not only directly initiates the demineralization of dental hard tissues but also, more critically, inhibits the critical remineralization function facilitated by saliva. Ultimately, this dynamic shifts the equilibrium between demineralization and remineralization toward a state of net mineral loss (i.e., in favor of demineralization) (Featherstone 1999, 2000). This imbalance constitutes the central pathological mechanism underlying dental caries development (Frencken et al. 2017) and ultimately leads to enamel demineralization and the formation of cavities (Anderson 2002; Strużycka 2014).

Although the best solution for dental caries is early prevention, effective treatment is also required to ensure that teeth can be used for a long time. Regarding the treatment of dental caries, Cabalén et al. (2022) argued that sealants, fluoride gels, and varnishes are effective strategies to prevent the occurrence of dental caries and the occurrence of dental caries at an early stage. The application of natural human antimicrobial peptides is also a potential treatment for dental caries. These substances can kill *S. mutans* to treat dental caries and also prevent dental caries by blocking the adhesion of bacteria (Niu et al. 2021; P. Hu et al. 2024; Ying et al. 2023). Nanodrug delivery systems will become a research hotspot in the near future, which will solve the various challenges of traditional drug delivery in oral application (Di Filippo et al. 2022; Abd‐Elsalam and Abouelatta 2023; Vanni et al. 2025). This new delivery system approach may significantly improve the treatment effect of dental caries (H. Du et al. 2025). While nano‐based drug delivery systems offer theoretical advantages—including enhanced solubility, controlled release, and targeted delivery—their clinical translation for oral diseases remains encumbered by substantial hurdles. The oral cavity presents multiple physiological barriers, such as salivary flow, the mucus layer, and fluctuating pH, all of which can compromise the stability and retention of nanoformulations (Kumari et al. 2025). Furthermore, concerns regarding long‐term biosafety and potential cytotoxicity remain inadequately addressed (Hobson et al. 2016; Patra et al. 2018). The high cost of nanomaterial production and the complexity of regulatory approval further impede their widespread clinical adoption (Xiong et al. 2023).

The cariogenic landscape is not static but evolves across the lifespan. While *S. mutans*, *Lactobacilli*, and *Actinomyces* emerge as conserved players across age groups, their relative abundance and ecological roles shift with host development, tooth eruption, and root exposure in the elderly. This age‐dependent microbial dynamics challenges the one‐size‐fits‐all paradigm and underscores the need for age‐stratified preventive strategies. Current evidence, however, remains largely cross‐sectional, leaving the temporal sequence of microbial succession—and the potential contribution of yet‐uncultivable species—poorly defined. Clinically, established measures such as sealants and fluoride remain the bedrock of caries prevention. Looking ahead, antimicrobial peptides and nanodrug delivery systems offer promising avenues for targeted intervention, but their clinical translation hinges on longitudinal validation and functional profiling to establish causality between specific microbial shifts and caries progression.

## 4 The Relationship Between Periodontal Disease and the Oral Microbiota

Periodontal disease, a condition affecting the supporting tissues of the teeth, appears in various forms, such as gingivitis and generalized periodontitis, which are among the most prevalent (Chapple et al. 2018; Heitz‐Mayfield 2024a; Villoria et al. 2024). Interestingly, differences in the incidence rates of periodontitis and gingivitis seem to exist according to age. It is widely believed that marginal gingivitis begins in childhood and tends to stabilize in adulthood (Page 1986). Meanwhile, it has become a consensus that chronic periodontitis mainly affects adults, although it cannot be denied that invasive periodontitis happens occasionally in children (Kinane et al. 2017). In people of all ages, gingivitis constitutes an initial inflammatory state of the gums that, if untreated, can progress to the destruction of periodontal tissues and noticeable attachment loss, leading to periodontitis (Heitz‐Mayfield 2024a; Newman and Socransky 1977; Neurath and Kesting 2024). Because gingivitis is regarded as an initial inflammatory state of the gums, there have been few separate studies on gingivitis in the past decade. Therefore, we will not discuss the microorganisms related to gingivitis separately, but instead analyze them together with those related to periodontal disease.

These infections caused by the microorganisms of gingivitis alter the periodontal ecosystem, promoting a disease state, as supported by Rosebury et al. (1950). The primary mechanisms at play here involve bacterial aggregation, plaque formation, and tissue degradation (Socransky 1970). Gram‐positive rod‐shaped oral *Actinomyces* are among the earliest colonizers of tooth surfaces, capable of forming aggregates with other early colonizing bacteria like *Streptococcus*, collectively constituting the foundation of early dental plaque, a key initiator of periodontal disease (Kolenbrander et al. 2006; Miranda et al. 2024; Manzoor et al. 2024).

**Figure 2:**
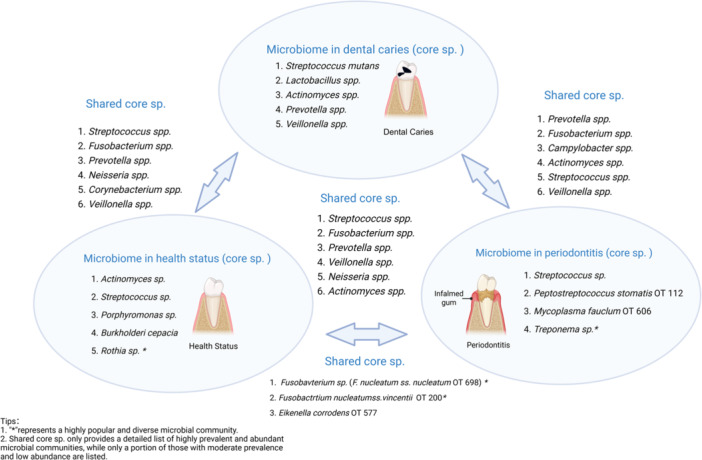
Comparison of oral microbial abundance among healthy people, patients with periodontitis, and individuals suffering from dental caries. The schematic image compares the abundance of different oral microorganisms among healthy people, patients with periodontitis, and individuals suffering from dental caries. The parts near the three arrows correspond to the microbiota shared by healthy people and patients with dental caries, patients with dental caries and periodontitis, and healthy people and patients with periodontitis, respectively. The middle part represents the oral microorganisms shared by the three populations. There are also graphs in the figure showing the visual comparison of similar conditions in healthy people's gums, periodontitis patients' gums, and caries. “*” represents the species closely related to health or periodontitis, which is judged according to the repeated occurrence in the molecular survey conducted by different groups in different patient groups. This figure was created in BioRender.

As we all know, some microorganisms exist in normal periodontal tissue, so periodontal disease is not entirely related to infection. The occurrence of periodontal disease may also result from an ecological imbalance to a certain extent. Periodontal disease stems from an environmental imbalance, characterized not only by shifts in pathogen numbers but also in pathogen proportions. Diao et al. (2021) identified a microbial dysbiosis gradient correlating with disease severity. Shifts in specific species like *Prevotella intermedia* and *Catonella morbi* have been proposed as potential periodontitis biomarkers. Hong et al. (2015) and Abusleme et al. (2013) employed 16S rRNA gene sequencing to differentiate oral bacteria in healthy individuals from those with periodontal disease, illustrating the most prevalent bacteria identified in the healthy subgingival crevice. The following figure intuitively illustrates the differences in oral microbiota abundance between healthy individuals and periodontitis patients (Figure 2).

One significant discovery from the characterization of the subgingival microbiome through 16S rRNA gene sequencing is the identification of species whose proportions remain consistent regardless of the individual's oral health status. These species, known as core species, are present in similar proportions regardless of whether the individual is in a state of health or disease (Curtis et al. 2020). Among these core species, *Campylobacter gracilis* and *Fusobacterium nucleatum* ss. *vincentii* are consistently detected.

Balta et al. (2021) concluded that certain monoclonal antibodies and statins can alleviate periodontitis. Recent studies have shown that probiotics also exert anti‐inflammatory effects and can reduce concentrations of inflammatory factors (Balta et al. 2021; Scannapieco and Gershovich 2020; Bodke and Jogdand 2022). Despite this promising finding, several limitations must be acknowledged. Notably, the effects of probiotics are strain‐specific, and optimal dosing regimens remain to be established. Furthermore, their safety in specific populations—such as immunocompromised individuals—requires rigorous evaluation. The current evidence base is also constrained by a lack of large‐scale, long‐term randomized controlled trials (RCTs) with adequate follow‐up to confirm efficacy. In addition, future studies should assess patient compliance and acceptability across diverse populations to facilitate the translation of probiotic therapies into clinical practice (Alasbily et al. 2025).

*Treponema denticola*, sharing helical morphology with the sexually transmitted pathogen *Treponema pallidum* (Y. Xiao, Liu, et al. 2016; W. Li et al. 2023), as both are found within the *Treponemataceae* family, has garnered increasing research attention. *T. denticola*, a recognized keystone pathogen in periodontal disease (Peng et al. 2022), often exists in the mouth and is considered to be composed of *Porphyromonas gingivalis* and *Tannerella forsythia*. Its major surface protein, a critical virulence factor, mediates microbial adhesion, modulates immune responses, facilitates pore formation, and contributes to various pathological changes in tissues and cells (Zhao et al. 2025). While principally considered a periodontal pathogen, recent investigations suggest a potential association between *T. denticola* and neurodegenerative disorders, including Alzheimer's disease (Pisani et al. 2023), and oral phosphorus cancer (Peng et al. 2022).

Traditional ecological imbalance theory focuses on the proportion of flora but ignores functional redundancy—that is, that different types of flora can perform similar pathogenic or metabolic roles. Therefore, it is important to integrate various theories for a more comprehensive analysis.

Owing to their pathophysiological resemblance to periodontitis (Eggert and Levin 2018; Salvi et al. 2023), the rising incidence of peri‐implant diseases accompanying the expansion of oral implantology warrants their inclusion in this discussion (Scarano et al. 2023). Peri‐implant diseases encompass two main entities: peri‐implant mucositis and peri‐implantitis (Klinge et al. 2018). The former refers to a reversible inflammatory lesion confined to the peri‐implant soft tissues, analogous to gingivitis (Heitz‐Mayfield and Salvi 2018). In contrast, peri‐implantitis is a destructive inflammatory condition affecting both soft and hard tissues, ultimately leading to progressive bone resorption and representing a primary cause of implant failure (Roccuzzo et al. 2023; Schwarz et al. 2018). Analogous to the relationship between gingivitis and periodontitis, peri‐implant mucositis is considered the precursor to peri‐implantitis (Heitz‐Mayfield 2024b). These two conditions partially share considerable overlap in their pathogenic microbial profiles, further supporting their analogous disease mechanisms. Scarano et al. (2023) concluded that while the microbial species colonizing healthy periodontal and peri‐implant tissues are similar to some extent, their microbial profiles diverge under pathological conditions; moreover, distinct microbial communities are also observed between healthy and diseased peri‐implant tissues. Specifically, peri‐implantitis was characterized by the abundance of *Capnocytophaga leadbetteri*, *Treponema maltophilum*, *Peptostreptococcus* spp., *Neisseria* spp., *P. gingivalis*, *Porphyromonas endodontalis*, *Lactococcus lactis*, and *Filifactor alocis* (Kensara et al. 2024). Recent studies have also implicated *Candida* and some viruses in peri‐implantitis, warranting further research to clarify their role in disease pathogenesis (Lafuente‐Ibáñez de Mendoza et al. 2021; Kensara et al. 2021). Complementing these emerging findings, a meta‐analysis conducted by Carvalho et al. (2023) demonstrated a significant association between peri‐implantitis and the presence of *Staphylococcus epidermidis* alongside several classic periodontopathogens: *P. gingivalis*, *T. forsythia*, *T. denticola*, *F. nucleatum*, and *P. intermedia*. Despite the heterogeneity in microbial profiles reported across studies—likely attributable to differences in detection methods, study populations, and disease definitions—the overlapping presence of key periodontopathogens in both periodontitis and peri‐implantitis underscores a partially shared etiological basis.

At its core, periodontal disease exemplifies ecological dysbiosis rather than a conventional infection by a single pathogen. The severity of disease tracks with compositional shifts—such as the enrichment of *P. intermedia* and *C. morbi*—while core species like *C. gracilis* and *F. nucleatum* ss. *vincentii* persists across health and disease states, suggesting that community structure, not merely pathogen presence, dictates clinical outcome. Despite this conceptual advance, mechanistic insights remain scarce: we know which microbes shift, but not how they functionally interact or how host‐specific responses modulate disease trajectory. This ecological paradigm extends to peri‐implant diseases, where overlapping periodontopathogens and unique species such as *S. epidermidis* shape disease pathogenesis, highlighting both shared and niche‐specific microbial drivers. Adjunctive therapies, including anti‐inflammatory agents and probiotics, show clinical benefit, yet robust randomized trials are lacking. Future research must pivot from compositional surveys to functional interaction networks and host–microbe crosstalk, paving the way for personalized periodontal and peri‐implant interventions tailored to individual microbial and immunological profiles.

## 5 The Link Between Dental Caries and Periodontal Disease

**Figure 3:**
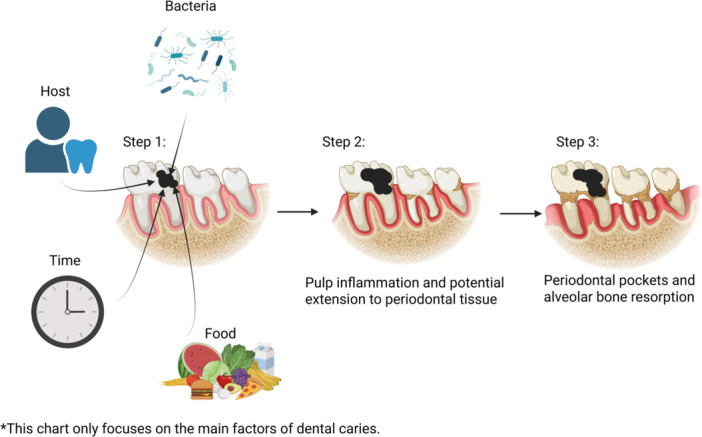
Progression of dental caries from initial damage to periodontal involvement. This schematic diagram combines the four‐factor theory of dental caries to explain how the initial caries damage leads to pulpitis. The black area represents the damaged area, which gradually expands over time. This figure was created in BioRender.

Some pathogenic microorganisms of dental caries and periodontal disease overlap (Figure 2). Considerable evidence supports the notion that dental caries and periodontal disease represent progressive destruction, with damage to periodontal tissue often challenging to prevent without early intervention in the caries' initial stages. Caries development hinges on the interplay of four key factors: food, host, bacteria, and time (X. Chen et al. 2020). *M. streptococci*, particularly *S. mutans*, as well as *Lactobacilli*, exhibit strong correlations with caries (Tanner et al. 2018; Ye et al. 2024; Sharma et al. 2024). *S. mutans* ferments sucrose and other sugars efficiently to generate adenosine triphosphate, with lactic acid serving as a byproduct (Zeng and Burne 2016). The accumulation of lactate leads to localized acidification of the caries environment (Guo et al. 2013; Hwang et al. 2016). This acid interacts with calcium hydroxyapatite, the primary component of tooth enamel, triggering enamel demineralization and the formation of superficial caries (Sampaio et al. 2024; Ibrahim et al. 2020). With time, superficial caries progresses to deep caries, enabling bacterial infiltration into the pulp with subsequent pulp inflammation and potential extension to periodontal tissue, leading to periodontal pockets and alveolar bone resorption (Figure 3). Timely detection of changes in tooth color indicative of caries formation and prompt intervention effectively halts periodontal destruction. However, in clinical practice, the relationship between dental caries and periodontal disease is more complex. Not all deep caries can lead to periodontitis, and its progress is also regulated by the immune ability, restorative treatment intervention, and other factors. Establishing a more precise explanation of the relationship between the two requires more clinical epidemiological data.

## 6 Salivary Gland Disease

Salivary gland inflammation and salivary gland calculus are among the most prevalent diseases affecting the salivary glands. Bacterial or viral infections can lead to salivary gland inflammation. Bacterial infections can develop into acute or chronic forms (M. J. Kim et al. 2024). Acute bacterial sialadenitis occurs when bacteria invade the salivary glands, often as a result of an ascending infection originating from the oral cavity (Carlson 2009; Danstrup et al. 2020). Chronic bacterial sialadenitis is typically associated with ductal obstruction caused by factors such as mucous plugs, strictures, or adhesions, and, in cases of juvenile chronic parotitis, malformations of the excretory ducts or congenital duct ectasia should be considered (Delli et al. 2014). Viral sialadenitis is commonly thought to include diseases such as mumps, giant cell inclusion body disease, and AIDS virus–associated salivary gland disease.

The pathogenesis of salivary gland diseases exemplifies a multistage continuum—from initial microbial colonization to biofilm formation, tissue damage, and in some cases, sialolith mineralization—rather than a single‐step infectious event. This process is governed by the dynamic interplay between microbial virulence traits and host defense factors that collectively determine disease initiation, clinical phenotype, and long‐term outcomes.

Initial colonization and adhesion represent the first critical step in microbial infection within the salivary gland duct system. To establish colonization, oral microorganisms must overcome the mechanical flushing effects of salivary flow and evade innate immune components. This process is primarily mediated by specific bacterial adhesins. For instance, *Streptococcus sanguinis* utilizes surface pili to bind salivary α‐amylase adsorbed onto the ductal epithelium, thereby facilitating persistent colonization (Okahashi et al. 2011). Similarly, the colonization of tooth surfaces by *Actinomycetes* and *Streptococci* involves bacterial adhesion to salivary components within the acquired enamel pellicle, a process also governed by specific adhesin–receptor interactions (Ruhl et al. 2004).

Biofilm formation then ensues, protecting bacteria from clearance and antimicrobials. Schrøder et al. (2018) observed the presence of bacterial biofilms in the submandibular gland tissues of patients with chronic obstructive sialadenitis, suggesting that biofilms may contribute to the initiation and progression of this condition.

Tissue injury results from both microbial factors and host inflammatory responses. For example, *Staphylococcus aureus* secretes hemolysins and proteases that disrupt epithelial integrity (X. Zhang et al. 2017; Kline et al. 2024). Additionally, host‐derived antimicrobial peptides (such as cathelicidin) may exacerbate inflammation by upregulating the expression of pro‐inflammatory genes (Popa et al. 2024). Although studies on such pro‐inflammatory mediators in the context of salivary gland diseases remain limited, it is plausible that similar mechanisms contribute to the pathogenesis of salivary gland inflammation—an area that warrants further investigation.

**Figure 4:**
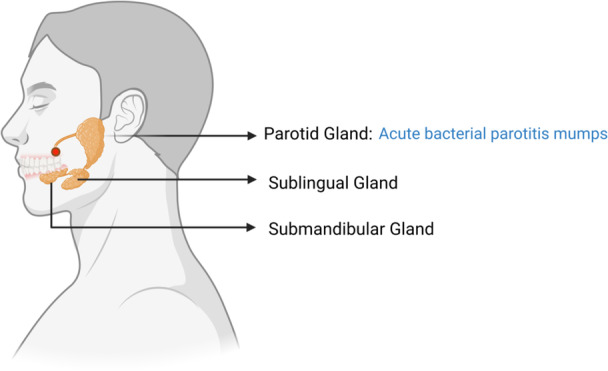
Schematic image of the anatomy of the large salivary gland and common diseases of the parotid gland. The three salivary glands are adjacent to each other, but their openings are very different. The red dot shows the approximate position of the parotid duct opening. This figure was created in BioRender.

Host anatomical and immune factors modulate each stage. The parotid gland is most commonly inflamed due to its anatomical and physiological traits. Its duct opening is situated farther from the tongue, increasing susceptibility to microbial invasion (M. J. Kim et al. 2024). Therefore, the opening of the parotid duct is more susceptible to invasion by microorganisms contained in saliva (Figure 4). Physiologically speaking, the saliva secreted by the parotid gland is completely serous, unlike that secreted by other glands. Mucinous fluids contain substances that can prevent bacterial infection, which is the reason why the parotid gland is more susceptible to bacterial infections (McQuone 1999).

Acute bacterial parotitis (ABP) can be categorized into hospital‐acquired and community‐acquired types. In hospital‐acquired ABP, *S. aureus* is prevalent in more than 50% of cases. At the same time, patients in intensive care units are often infected by hospital‐acquired flora such as *Eikenella corrodens, Escherichia coli, Fusobacterium* spp., *Klebsiella* spp., *Prevotella* spp., and/or *Pseudomonas* spp. Community‐acquired ABP is more frequently diagnosed than the hospital‐acquired form and is typically associated with *S. epidermidis* and *Streptococcus* spp. (Carlson 2009; Nicolasora et al. 2009). Carlson's research identified two types of chronic bacterial parotitis (Carlson 2009). The adult type is usually associated with *S. aureus*, while the juvenile type is frequently linked to *Viridans streptococci*.

Mumps stands out as the most prevalent viral disease affecting the salivary glands, characterized by acute bilateral nonsuppurative swelling of the parotid glands, although all salivary glands can be affected (Schreiber and Hershman 2009; Katoh et al. 2024). This disease is caused by a single‐stranded *Paramyxovirus* with a particular affinity for glandular epithelium (Takahashi et al. 2024). Viral replication in the parotid duct epithelium, along with the infiltration of lymphocytes and macrophages, results in periductal interstitial edema and local inflammation (Delli et al. 2014). Additionally, influenza, parainfluenza, coxsackie, echo, and lymphocytic choriomeningitis viruses have been implicated in multiple episodes of mumps (Bradley 2002; Silvers and Som 1998).

In recent years, increasing attention has been directed toward the relationship between xerostomia in Sjögren syndrome and salivary gland infection. Although Sjögren syndrome is widely recognized as an autoimmune disorder with diverse systemic manifestations (Negrini et al. 2022), viral infection has been implicated as a potential pathogenic factor. Maldonado et al. (2022) suggested that hepatitis C virus (HCV) infection alters salivary gland histology and salivary composition, leading to changes in markers of acinar and ductal function, thereby contributing to the development of xerostomia. In the absence of hepatitis B virus (HBV) coinfection, HBV has been detected in minor salivary glands of patients with Sjögren syndrome, suggesting a possible role for HBV in the pathogenesis of salivary gland lesions. However, relevant studies remain limited, and further investigation is warranted (Hesterman et al. 2023). Human T‑cell leukemia virus type 1 (HTLV‑1) has also been proposed as a potential etiological agent in Sjögren syndrome, with evidence indicating that it may spread to salivary gland epithelial cells via unique biofilm‑like structures (Nakamura et al. 2022). Research on salivary adenovirus infection has drawn attention to its possible link with Sjögren syndrome, which may offer new insights toward curative strategies for the disease.

Sialolithiasis is the most common disorder affecting the salivary glands in middle‐aged patients, leading to recurrent swelling of the salivary gland and chronic obstructive sialadenitis (Teymoortash et al. 2002). Dental calculi originate from nonmineralized biofilms (dental plaque) that capture various oral bacterial species, human proteins, food debris, and significant amounts of mineral salts from saliva. During this process, microorganisms enclosed in the self‐produced matrix become trapped in the calcified mass (Akcalı and Lang 2018). The core flora found in salivary stones includes *Streptococcus* spp., *Fusobacterium* spp., *Eikenella* spp., *Campylobacter* spp., *Actinomyces* spp., *Kingella* spp., *Neisseria* spp., *Prevotella* spp., and *Haemophilus* spp., which constitute the predominant oral microorganisms in the core microbiota of the salivary calculi. Analysis of the correlations among the microorganisms forming the calculi microbiota revealed *Fusobacterium* spp., *Streptococcus* spp., and *Kingella* spp. as the key bacterial nodes, with *Fusobacterium* spp. identified as the central hub due to its ability to aggregate with numerous bacterial species involved in biofilm formation (Edwards et al. 2007).

Conservative treatment strategies for salivary gland diseases are usually based on multidimensional interventions, including etiological treatment and incentive regulation (such as pain management and basic disease control) and combined salivary flow stimulation measures (salivary secretion stimulant application, systematic hydration support, and local physical intervention such as gland massage and hot compress), supplemented by oral health enhancement and drug intervention scheme adjustment. In addition, when it comes to salivary gland calculi, the use of salivary gland endoscopy can be considered. As a minimally invasive gland‐preservation therapy, this procedure can be extended to the precise intervention of obstructive and nonobstructive salivary gland diseases through the exploration of the endogenous ductal system and pathological removal, and can achieve etiological targeted therapy while maintaining the integrity of gland function. However, it has not been widely applied to date in oral and maxillofacial surgery (M. J. Kim et al. 2024; G. Zhu and Li 2023). Recently, it has been found that exosomes also have a specific therapeutic effect in salivary gland diseases, especially in salivary gland calculi (Cui et al. 2025). However, their clinical translation faces substantial hurdles. First, regulatory frameworks remain nascent: no exosome products have received global marketing approval, and standards vary across regions, requiring rigorous safety and efficacy data (Verma and Arora 2025). Second, production standardization is lacking, including the need for improved methods for large‐scale exosome production (Kostyusheva et al. 2025). Third, mechanisms of action are incompletely understood: exosome cargo varies with parent cell status, and data on biodistribution, and targeting efficiency remain extremely limited (Abbasi et al. 2025; X. Liu et al. 2025). As recent reviews emphasize, standardized quality control systems and rigorous preclinical and clinical validation are urgently needed before clinical application.

Salivary gland diseases present a dual etiological landscape. Broadly, these can be categorized into bacterial infections, which predominantly drive acute and chronic sialadenitis, with *S. aureus* and *Streptococcus* as the primary pathogens. Notably, while the common mumps virus is a classic cause of acute viral sialadenitis, other viruses such as HCV, HBV, and HTLV‐1 have been of particular research interest due to their potential association with Sjögren syndrome. The pathogenesis follows a multistage continuum—from initial bacterial adhesion and colonization, to biofilm formation within obstructed ducts, tissue injury driven by microbial toxins and host inflammatory responses, and ultimately, in sialolithiasis, mineralization of bacterial aggregates into calculi. Host anatomical factors further modulate susceptibility: the parotid gland's serous secretions and ductal anatomy predispose it to ascending infection. Yet, despite this mechanistic understanding, the dynamics of the salivary microbiome during active infection and in response to treatment remain largely uncharted. On the therapeutic front, conservative measures and culture‐guided antibiotics remain mainstays for acute infections. For obstructive disease, sialendoscopy offers a minimally invasive, gland‐preserving option, though its adoption remains limited. Emerging exosome‐based therapies hold promise for restoring glandular function, but rigorous investigations are needed to establish efficacy. Future research should prioritize longitudinal microbiome profiling, mechanistic studies using organoid models, and translational efforts to accelerate novel regenerative therapies.

## 7 Oral Mucosal Lesions

**Figure 5:**
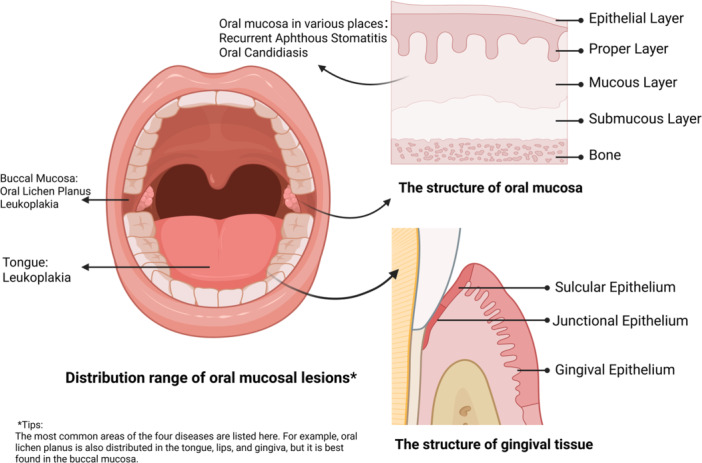
Distribution range of oral mucosal lesions and schematic diagram of the gingival and oral mucosa structure. This figure was created in BioRender.

Common oral mucosal lesions include oral lichen planus (OLP), leukoplakia, recurrent aphthous stomatitis (RAS), and oral candidiasis (Carrozzo et al. 2019; Cammarata‐Scalisi et al. 2020; Randall et al. 2022; Hellstein and Marek 2019). Their predilection sites in the mouth are different (Figure 5), and the microbial species related to these oral mucosal diseases vary (Table 1).

**Table 1:** Pathogenic microorganisms and their mechanisms in common oral diseases.

Disease	Key associated microorganisms	Evidence type	Key pathogenic mechanisms
Dental caries	*Streptococcus mutans, Lactobacillus*	Clinical (cross‐sectional)	Acid production
	*Scardovia wiggsiae* (children)		Age‐dependent ecology
	*Propionibacterium acidifaciens*, *Olsenella profuse* (elderly root caries)		
Periodontal disease	*Porphyromonas gingivalis*, *Tannerella forsythia*	Clinical (16S sequencing)	Ecological dysbiosis; functional redundancy
	*Treponema denticola*		
	Core species (*Campylobacter gracilis*,		
	*Fusobacterium nucleatum* ss. *vincentii*)		
Salivary gland diseases	*Staphylococcus aureus, Streptococcus* (sialadenitis)	Clinical (16S sequencing)	Biofilm formation; mineralization
	*Fusobacterium* spp. (sialolithiasis)		
OLP	EOLP: ↑*Candida*, *Aggregatibacter*, *Lactobacillus*	Clinical (mycobiome)	Interkingdom dysbiosis
	NEOLP: ↑*Aspergillaceae, Prevotella intermedia*		
Leukoplakia	↑*Fusobacterium*, ↑*Leptotrichia*	Clinical	Reduced diversity; early warning
	Animal: Shifts precede symptoms	Animal	
RAS	↑*Acinetobacter johnsonii* (risk)	Clinical (model)	Dysbiosis index (83% predictive)
	↓*Streptococcus salivarius* (protective)		
Oral candidiasis	*Candida albicans*	Clinical	Interkingdom interactions
	NACS (*C. glabrata* and *C. krusei*)	In vitro	Azole resistance
Oral cancer	*P. gingivalis*	In vitro/animal	5 mechanisms
	*C. albicans*, HPV	Clinical	Need human validation

Abbreviations: EOLP, erosive oral lichen planus; HPV, human papillomavirus; NACS, non‐*albicans Candida* species; NEOLP, nonerosive oral lichen planus; OLP, oral lichen planus; RAS, recurrent aphthous stomatitis.

### 7.1 OLP

OLP is a common chronic inflammatory disease of the oral mucosa. It is clinically categorized into erosive OLP (EOLP) and nonerosive OLP (NEOLP) forms (Beibei et al. 2024), and predominantly affects women between the ages of 30 and 60 years. The reticular and erosive forms of this condition are most common and can cause significant pain and discomfort (J. Chen et al. 2022). Of note, OLP is a chronic inflammatory condition mediated by T‐cells. Although its etiology and pathogenesis remain poorly understood, they likely involve multiple factors such as infections, autoimmunity, stress, and medications (Mostafa and Tarakji 2015; C. Lavanya and Ranganathan 2024; Ślebioda et al. 2024). Additionally, oral hygiene practices, including plaque and calculus accumulation, can exacerbate the onset and progression of OLP (Crincoli et al. 2011). Topical corticosteroids are the first‐line treatment for OLP due to their ability to modulate inflammation and immune responses by reducing lymphocytic exudate and stabilizing the lysosomal membrane (N. Lavanya et al. 2011). Backman and Jontell also demonstrated that chlorhexidine treatment significantly alleviates the symptoms of lichenoid lesions (Bäckman and Jontell 2007).

Oral bacterial dysbiosis has been observed in patients with OLP (Tuominen and Rautava 2021). In a study by J. Chen et al. (2022) noted that compared to the healthy group, the abundance of *Neisseria, Haemophilus, Fusobacterium, Porphyromonas, Rothia, Actinomyces*, and *Capnocytophaga* was significantly increased in the OLP group. However, existing literature presents conflicting results regarding the distribution of *Neisseria* and *Haemophilus* (J. Chen et al. 2022), with some studies reporting contradictory findings (K. Wang et al. 2016; Deng et al. 2020; G. Du et al. 2020). Discrepancies among researchers may stem from variations in ethnic groups, diagnostic criteria, and analytical methods, which can impact study outcomes. *Actinomyces* levels were elevated in male OLP patients, while those of *Neisseria, Haemophilus, Porphyromonas, Fusobacterium, Rothia*, and *Capnocytophaga* were increased in female OLP patients. Further, in female OLP patients, *Lautropia* and *Dialister* exhibited a positive correlation with age, while *Moraxella, Porphyromonas*, and *Fusobacterium* showed a positive correlation with age in male OLP patients (J. Chen et al. 2022), suggesting that age and gender influence OLP‐associated microbes. Hormones may represent a crucial factor contributing to these findings. Alteration in the oral microbiota in the absence of estrogen, coupled with decreased estrogen levels in perimenopausal women, may predispose individuals to OLP (Mohan et al. 2017). Furthermore, age‐related declines in immune function, known as “immunosenescence,” could also contribute to the greater susceptibility of elderly individuals to microbial infections (Feres et al. 2016).

Significant differences in microbial composition exist between subtypes of OLP. Both EOLP and NEOLP exhibit distinct degrees of fungal and bacterial dysbiosis. In EOLP, fungal α‐diversity is low, with *Candida* as the predominant genus, while bacterial profiles show significantly lower abundance of *Streptococcus* alongside enrichment of *Aggregatibacter* and *Lactobacillus*. In contrast, NEOLP is characterized by significant enrichment of *Aspergillaceae* and *P. intermedia*. Beyond compositional shifts, the microbial co‐occurrence and co‐exclusion networks show distinct patterns across OLP subtypes. In EOLP, *Lactobacillus* assumes a key bridging role within the microbial community, suggesting that interkingdom interactions—rather than the effects of individual species—may contribute to disease pathogenesis and shape clinical phenotypes (Beibei et al. 2024).

### 7.2 Leukoplakia

“White plaques that exclude the suspected risk of (other) known diseases or diseases that do not increase the risk of cancer”—this is the definition of leukoplakia (Warnakulasuriya et al. 2007). Around 70% of oral leukoplakia (OLK) lesions manifest on the buccal mucosa, lip vermilion, and gingivae (Mortazavi et al. 2014).

Longitudinal studies in animal models of OLK have revealed that shifts in oral microbial diversity precede the manifestation of clinical symptoms. Using rat OLK models and 2bRAD‐M sequencing, researchers observed elevated abundances of *Streptococcus*, *Glaesserella*, and *Pseudomonas aeruginosa* during early disease stages, suggesting that microbial dysbiosis may serve as an early warning signal for malignant transformation (Sang et al. 2025).

Smoking promotes leukoplakia via macrophage polarization and glutamine metabolism (Y. Zhu, Zhang, et al. 2021). M. Li et al. (2022) also implicated increases in lipid peroxides and interleukin‐6 in its progression. The formation of OLK does not seem to have much to do with the involvement of microorganisms. However, we can still find that the microbial species in individuals with OLK differ from those seen in individuals with normal mucosa. In patients with leukoplakia, bacterial richness and diversity tend to be reduced compared to in healthy controls (Pietrobon et al. 2021). *Haemophilus, Leptotrichia, Campylobacter, Rothia mucilaginosa, Bacteroidetes, TM7* (also known as *Saccharibacteria*), *P. gingivalis, Prevotella*, and *Fusobacteria* are more prevalent in leukoplakia, while *Firmicutes* levels are less common (Pietrobon et al. 2021; X. Hu et al. 2016; Amer et al. 2017; Shridhar et al. 2021; Pignatelli et al. 2024).

Proliferative verrucous leukoplakia, an aggressive subtype with high malignant transformation risk, exhibits pronounced dysbiosis characterized by enrichment of *Fusobacterium* and *Porphyromonas*. These taxa contribute to biofilm formation, immune evasion, and modulation of epithelial signaling pathways, potentially synergizing with host genetic and epigenetic factors to drive oncogenic progression (Špiljak et al. 2025).

Hosmani et al. (2021) proposed that recombinant adenovirus p‐53 can be used to treat OLK, which has the potential for a beneficial therapeutic effect, but further clinical trials with more patients are needed because its use may cause fever and other symptoms. Photodynamic therapy, a minimally invasive treatment, can also be used; in particular, its combination with a nanostructured drug delivery system can solve the limitation of photosensitizer application so that it can be used to treat OLK and other oral precancerous lesions (Angjelova et al. 2023).

### 7.3 RAS

RAS is the most prevalent oral mucosal disease in the general population. It is characterized by multiple recurrent round or ovoid inflammatory ulcerations with well‐defined margins that are surrounded by erythematous haloes, and displaying yellow or gray floors (Jurge et al. 2006). RAS causes significant pain, disrupts oral functions (eating, speech, and toothbrushing), and consequently impacts quality of life (Al‐Omiri et al. 2015). Various factors, including genetic predisposition, immunologic disturbances, viral and bacterial infections, food allergies, deficiencies in vitamins and microelements, systemic diseases, hormonal imbalances, mechanical injuries, and stress, have been implicated as triggers or having associations with RAS (Ślebioda et al. 2014). However, the exact etiology of RAS remains unclear. The treatment of oral aphthous ulcers involves both topical and systemic agents, with topical corticosteroids serving as the cornerstone of therapy (Gasmi Benahmed et al. 2021).

A study revealed that at the phylum level, *Proteobacteria* are significantly more abundant in the oral microbiota of individuals with RAS than healthy individuals. Additionally, the family *Paraprevotellaceae* and its genus *Prevotella* were notably enriched in the oral microbiota of those with RAS. Conversely, *Firmicutes* were significantly reduced in the oral microbiota of RAS patients. In comparison to healthy individuals, microorganisms of the class *Bacilli* and its derivatives (order *Gemellales*, family *Gemellaceae*, and order *Bacillales*, family *Paenibacillaceae*) were found at decreased levels in individuals with RAS. Furthermore, the reduced phylotypes in RAS include the genus *Anaerovorax* and family *Xanthomonadaceae* (Z. Zhu, He, et al. 2021). Yang et al. (2020) previously suggested that the occurrence of RAS is significantly associated with an increase in *E. coli* and *Alloprevotella*, along with a decrease in *Streptococcus*. Similarly, Y. Kim et al. (2016) concluded that RAS is linked to dysbiosis in the mucosal and salivary microbiota, with reduced levels of *Streptococcus salivarius* and increased levels of *Acinetobacter johnsonii* in the mucosa linked to RAS risk. However, these findings must be interpreted with caution because establishing causal relationships in the design of cross‐sectional studies is challenging. Some literature only shows a correlation between microbial communities and RAS. Still, it is impossible to determine whether the observed dysbacteriosis is the cause of the ulcer or a secondary consequence after the ulcer occurs. More prospective cohort studies and interventional experiments are needed to clarify the exact causal relationship in the future.

At present, the radical treatment of RAS has not been reported (Conejero Del Mazo et al. 2023), but, as it is a kind of stomatitis, local corticosteroids are its first line of treatment (Gasmi Benahmed et al. 2021).

### 7.4 Oral Candidiasis

Oral candidiasis caused by *Candida* infection is a common head and neck disease. Many factors can cause oral candidiasis, including both local and systemic host factors; local factors include wearing dentures, impaired salivary gland function, inhaled steroids, and oral cancer, while systemic factors include extremes of age, smoking, diabetes mellitus, Cushing's syndrome, immunosuppression, malignancies, nutritional deficiencies, and antibiotics. Wearing dentures produces a microenvironment conducive to the growth of *Candida* with low oxygen, low pH, and an anaerobic environment. Other factors include oral cancer/leukoplakia and a high‐carbohydrate diet (Akpan and Morgan 2002; Jørgensen 2024).

The pathogenesis of oral candidiasis depends critically on local microenvironmental factors. Saliva plays a central role in maintaining *Candida* commensalism through: mechanical clearance via salivary flow; antimicrobial peptides (histatins, lactoferrin, and lysozyme); and competition with bacterial commensals. Disruption of these protective mechanisms—whether by xerostomia (medication‐induced), or broad‐spectrum antibiotics (eliminating bacterial competitors)—shifts the balance toward fungal overgrowth and tissue invasion (Jørgensen 2024; Vila et al. 2020).

*Candida* in the oral cavity exists as a fungus in the form of yeast (circular fungi), mold (filamentous fungi), or a combination of both (dimorphic fungi), with the most common type being *Candida albicans* (Singh et al. 2014). The ligands on the oral mucosa mediate the endocytosis of *Candida* and then promote the expression of down‐regulated protein 9 signaling. *C. albicans* expresses proteases, phospholipases, and lipases that promote the injury of epithelial cells and the occurrence of inflammatory reactions (Lass‐Flörl et al. 2024; Lopes and Lionakis 2022).

While *C. albicans* remains the predominant cause of oral candidiasis, non‐*albicans Candida* species (NACS), including *C. glabrata*, *C. krusei*, *C. tropicalis*, and *C. dubliniensis*, have emerged as significant pathogens, partly due to their intrinsic or acquired resistance to azole antifungals. These species vary considerably in virulence traits: *C. glabrata* lacks hyphal formation but expresses EPA adhesins; *C. dubliniensis* produces pseudohyphae and biofilms (Jørgensen 2024).

As a disease caused by fungi, antifungal treatment of oral candidiasis is a common approach (Quindós et al. 2019). Nystatin and miconazole are two common local antifungal drugs that have significant therapeutic effects on oral candidiasis, but require long‐term use to achieve eradication (Quindós et al. 2019). In addition, antimicrobial photodynamic therapy as a novel strategy for treating oral candidiasis has received attention from researchers in the past decade. Its advantages include highly selective bactericidal activity and no damage to oral tissue, making it a promising alternative treatment method. Despite its potential, clinical data on this method are sparse, and a well‐characterized understanding of its underlying mechanisms and efficacy is currently absent, representing a substantial gap that warrants future research (Janeth Rimachi Hidalgo et al. 2019; de Souto Medeiros et al. 2023; Mima et al. 2023). In terms of prevention, people must avoid the pathogenic factors mentioned above as much as possible. Identifying and eliminating any potential host susceptible factors is the basic principle for the management of candidiasis (Lewis and Williams 2017).

OLP, leukoplakia, RAS, and oral candidiasis exhibit distinct microbial profiles. Nevertheless, discrepancies persist in the literature due to variations in sampling techniques and demographic variables. The etiology of certain oral mucosal diseases remains incompletely elucidated, with hormonal and immune factors contributing to their complexity. Treatment approaches differ depending on the specific condition, yet they frequently result in high recurrence rates and adverse effects. To enhance our comprehension of these disorders, forthcoming investigations should prioritize prospective studies to elucidate causal relationships between microbial alterations and disease progression, as well as to develop more efficacious therapeutic strategies.

## 8 Oral Cancer

Malignant tumors remain a leading global cause of death, with their incidence expected to rise (Kiri and Ryba 2024; Filho et al. 2025). Among them, oral cancer accounts for about 3%–10% of the global cancer mortality profile, and its rising incidence rate deserves attention (Popovici and Ozon 2024). Oral squamous cell carcinoma (OSCC) is the most prevalent oral malignancy. It can manifest in any part of the oral mucosa, with the tongue, floor of the mouth, and gums being the most common sites (Sami et al. 2020). Most oral cancers are attributed to betel quid, smoking, and alcohol, with diet, hygiene, and genetics also contributing in some way (Johnson 2001; C. Li et al. 2024). Notably, around 15% of oral cancers are linked to infections involving viruses (like Human Papilloma Virus and Epstein Barr Virus), fungi like *C. albicans*, and certain bacteria (Chocolatewala et al. 2010). *Helicobacter pylori* is considered one of the microorganisms most relevant to the initiation and progression of human tumors. It serves as a crucial pathogenic factor in chronic gastritis, gastric ulcers, lymphomas associated with the lymphoid tissue of the gastric mucosa, and gastric adenocarcinomas (Karpiński 2019). Additionally, some studies suggest a potential link between *Salmonella typhi* and gallbladder cancer (Vaishnavi et al. 2005), between *Streptococcus bovis* and colon cancer (Gold et al. 2004), and between *Chlamydophila pneumoniae* and lung cancer (Littman et al. 2004). The exact mechanism of microbial influence on oral cancer remains unknown. Still, the impact of microorganisms on the progression of oral cancer can be inferred from the associations observed between cancers in other parts of the human body and microorganisms. In the attached table, the possible association mechanism between oral microbiome and oral cancer is more intuitively summarized (Table S1). However, most of the carcinogenic mechanisms reviewed in this section are derived from in vitro cell experiments or animal models, and their relative significance in the development of human oral cancer still needs to be validated. The oral cavity is a complex ecosystem, and the effect of a single microorganism may be enhanced or suppressed within the community environment. Future research should incorporate multiomics data to analyze how the microbial community as a whole collaborates to promote the carcinogenesis process under conditions that more closely resemble the human environment (such as an organ‐like model).

### 8.1 Chronic Inflammation

Chronic inflammation, often triggered by infections, is a key factor in cancer development and metastasis (Eslami‐S et al. 2020; Park et al. 2024). Microorganisms and their byproducts activate inflammatory cells, fibroblasts, and epithelial cells, stimulating the production of reactive oxygen species such as hydrogen peroxide and oxygen radicals, reactive nitrogen species like nitric oxide, active lipids and metabolites (such as malondialdehyde and hydroxy‐2‐nonenal) (Pushalkar et al. 2012). Elevated levels of specific pro‐inflammatory and pro‐angiogenic cytokines have been observed in the serum, saliva, and tissue samples of patients with oral cancer (Karpiński 2019; Issrani et al. 2022). Pro‐inflammatory cytokines play a pivotal role in the activation of key transcription factors within precancerous lesions, such as signal transducer and transcription activator 3 (STAT3) or nuclear factor (NF)‐κB. This activation facilitates increased cell proliferation, mutagenesis, oncogene activation, and angiogenesis, ultimately disrupting normal growth‐control mechanisms and leading to cancer development. Active substances or cytokines produced by immune cells can also directly initiate carcinogenesis by inducing mutations, genomic instability, or epigenetic changes (Vyhnalova et al. 2021; Grivennikov et al. 2010; Landskron et al. 2014).

### 8.2 Affecting the Cell Cycle, Promoting Cell Proliferation

Microorganisms can activate pathways like MAPK and cyclin D1, promoting cell proliferation and DNA replication. This disruption of the cell cycle can increase the rate of tumor gene mutations or directly induce mutations in tumor‐suppressor genes and proto‐oncogenes, thereby impacting tumor growth signaling pathways (Deo and Deshmukh 2020; Faden 2016; Coussens and Werb 2002).

### 8.3 Inhibiting Apoptosis

Microorganisms can inhibit apoptosis through antiapoptotic pathways, aiding cancer survival (Issrani et al. 2022; Vyhnalova et al. 2021). The intracellular accumulation of pathogens can suppress apoptosis primarily through the modulation of Bcl‐2 family proteins or by inactivating retinoblastoma protein (Nougayrède et al. 2005). Subsequently, partially transformed cells can evade self‐destructive processes and progress to a more advanced state of transformation, ultimately becoming tumorigenic (Chocolatewala et al. 2010). Notably, *P. gingivalis* activates Jak1/Akt/Stat3 signaling, which regulates intrinsic mitochondrial apoptosis pathways (Yilmaz et al. 2004; Mao et al. 2007). At the mitochondrial membrane, the proapoptotic activity of Bad is inhibited, leading to an increase in the ratio of Bcl‐2 (antiapoptotic) to Bax (proapoptotic), which subsequently limits the release of the apoptosis effector cytochrome *c* (Mao et al. 2007). Downstream, activation of both caspase‐9 and the executioner caspase‐3 is hindered (Yao et al. 2010). Another mechanism through which *P. gingivalis* contributes to the inhibition of cell apoptosis is by upregulating miRNA‐203, which, by downregulating suppressor of cytokine signaling 3, enhances the activity of STAT3, thereby inhibiting apoptosis (Vyhnalova et al. 2021; Moffatt and Lamont 2011).

### 8.4 Production of Carcinogens

Furthermore, certain oral pathogens produce volatile sulfur compounds such as hydrogen sulfide and methyl mercaptan. Even at low concentrations, these compounds exhibit toxicity to tissues and contribute to the pathogenesis of periodontitis and chronic inflammation (Milella 2015). H_2_S, a known genotoxic agent, can induce genomic instability and cumulative mutations (Attene‐Ramos et al. 2006). H_2_S can impact tumor growth and metastasis by activating proliferation, migration, and invasive signaling pathways, along with enhancing tumor angiogenesis (Hellmich and Szabo 2015). Lipopolysaccharide, a pathogenic substance commonly produced by many anaerobic oral bacteria, is known for its ability to trigger inflammatory processes, which are closely associated with the pathogenesis of inflammation‐related cancers. The stimulation of lipopolysaccharide during oral infections leads to elevated levels of cancer‐associated cytokines such as interleukin‐1β, interleukin‐6, and tumor necrosis factor‐α (Kang and Shin 2015). Numerous oral bacteria, including the genera *Lactobacillus, Lactococcus, Bifidobacterium, Streptococcus, Leuconostoc*, and *Pediococcus*, have the potential to produce lactic acid (Karpiński and Szkaradkiewicz 2013). The production of lactic acid influences the pH reduction in the local environment (Senneby et al. 2017). Particular species can generate various acids, such as the *aciduric Peptostreptococcus stomatis*, which produces acetic, butyric, isobutyric, isovaleric, and isocaproic acids (Downes and Wade 2006). The production of these acids may contribute to the acidic and hypoxic microenvironment of tumors, thereby enhancing metastatic efficiency (Lunt et al. 2009; Mazzio et al. 2010). Acetaldehyde, associated with alcohol consumption, has been classified as a Group I carcinogen by the International Agency for Research on Cancer (Perera et al. 2016). The microflora present in the tumor microenvironment, such as *Streptococci* (*Streptococcus gordonii, mitis, oralis, salivarius, sanguinis*) (Pavlova et al. 2013), and *Candida yeasts*, possess the enzyme alcohol dehydrogenase, which metabolizes alcohol into acetaldehyde (Marttila et al. 2013). This enzymatic activity may contribute to tumorigenesis by converting ethanol into acetaldehyde, a carcinogenic derivative capable of inducing DNA damage, mutagenesis, and secondary hyperproliferation of the epithelium (Deo and Deshmukh 2020). Smoking is also associated with cancer and leads to increased salivary acetaldehyde concentrations, thereby compounding the risk associated with alcohol consumption. The combined effect of smoking and alcohol consumption is synergistic (Meurman 2010; A. K. Gupta et al. 2022). Microbial carcinogenesis may involve nitrosation, wherein microbes produce N‐nitroso compounds from nitrites, amines, and amides. Various filamentous fungi, yeasts, and bacterial species, including common ones like *E. coli*, can catalyze nitrosation, with the production of carcinogenic nitrosamines being particularly linked to cancer development in the oral cavity (Faden 2016).

### 8.5 Cellular Invasion

*P. gingivalis* infection triggers the activation of the ERK 1/2‐Ets 1, p38/HSP27, and PAR2/NF‐κB pathways. This activation leads to upregulation of pro‐matrix metalloproteinase (MMP)‐9 (Inaba et al. 2014). These enzymes not only interact with the PAR2 receptor but also cleave the MMP‐9 proenzyme into its mature active form. MMP‐9 facilitates the degradation of the basement membrane and extracellular matrix, promoting the migration and invasion of cancer cells. This process enables carcinoma cells to infiltrate the lymphatic system and blood vessels, facilitating spread and metastatic growth at distant sites (Whitmore and Lamont 2014; Saba et al. 2024).

### 8.6 Clinical Evidence from Human Studies: From Association to Prediction

While the mechanistic insights discussed above are largely derived from in vitro and animal models, a growing body of clinical and longitudinal studies in humans is beginning to bridge the gap between microbial associations and clinically actionable predictions.

Prospective cohort studies have established that oral microbial dysbiosis precedes and predicts the development of oral cancer. Currently, the diagnosis of OSCC primarily relies on histopathological grading (Abati et al. 2020); however, this approach falls short in enabling early prediction or diagnosis of the disease. Radaic et al. suggested exploring biomarkers through genomics, transcriptomics, proteomics, metabolomics, immunohistochemistry, and microbiomics to enhance the reliability, sensitivity, and specificity of OSCC diagnosis. Nevertheless, discrepancies in the current literature highlight the need for further research to standardize these methodologies (Radaic et al. 2024; Siak et al. 2021; Khurshid et al. 2018).

Beyond cancer initiation, the oral microbiome also influences disease progression and prognosis. Certain members of the oral microbiota have been implicated in promoting tumorigenic functions associated with cancer development. For instance, *P. gingivalis* infection has been linked to oral digestive cancers and has been shown to enhance the invasion of oral cancer cells and promote the proliferation of oral cancer stem cells (Tuominen and Rautava 2021; Irfan et al. 2020; Lamont et al. 2022; Saikia et al. 2023). However, these findings are primarily associational, and further research is needed to establish a direct causal relationship. In addition, specific microbiota can significantly modulate responses to and the toxicity of various cancer therapies by directly interacting with the host, thereby influencing disease progression (Yusuf et al. 2023; Patil et al. 2023). In recent years, bacteria such as *S. typhi* and *Clostridium* species have been explored as potential carriers in targeted therapeutic strategies, offering a promising avenue for future research in oral cancer treatment (Irfan et al. 2020).

Longitudinal studies during cancer treatment further reveal dynamic host–microbe interactions. Longitudinal studies conducted during cancer treatment have provided valuable insight into the dynamic interactions between the host and the oral microbiome. L. Zhang et al. (2024) profiled the oral microbiota of 142 patients with head and neck cancer at multiple time points before, during, and after treatment, identifying four distinct trajectories of oral mucositis severity. Specific taxa, including *Prevotellaceae*, *Bacteroidota*, and *Proteobacteria*, showed significant correlations with mucositis severity over time, pointing to the potential for microbiome‐based strategies to personalize symptom management.

Multiple microorganisms—including *P. gingivalis*, *F. nucleatum*, *C. albicans*, and HPV—have been implicated in oral carcinogenesis through mechanisms involving chronic inflammation, cell cycle dysregulation, apoptosis inhibition, carcinogen production, and tissue invasion. However, the majority of mechanistic evidence remains derived from in vitro or animal models, which limits its direct translational relevance to human disease. Encouragingly, emerging human studies are beginning to address this gap: prospective cohort studies have linked specific microbial signatures to future oral cancer risk, while longitudinal investigations during cancer treatment have revealed dynamic associations between microbiome shifts and clinical outcomes such as the severity of oral mucositis. Despite this progress, critical challenges persist—including the unresolved causal hierarchy among microbial mechanisms, the poorly characterized temporal sequence of dysbiosis during malignant transformation, and the lack of standardized methodologies across studies. Future research should prioritize integrating multiomics approaches with human‐relevant organoid models to more accurately recapitulate the tumor microenvironment, alongside prospective surveillance of high‐risk populations and RCTs to assess whether microbiome‐targeted interventions can reduce cancer incidence or enhance therapeutic outcomes. Only through such coordinated efforts can the oral microbiome evolve from a descriptive biomarker into an actionable target for cancer prevention and personalized therapy.

## 9 Conclusion

Evidence increasingly shows that oral microorganisms significantly influence oral health and disease pathogenesis, with distinct microbial profiles for different diseases. Assessing the species and abundances of these microorganisms is crucial for developing effective preventive strategies against oral diseases. The attached table summarizes the pathogenic microorganisms and their mechanisms of action of common oral diseases in this review, which can provide valuable resources for further research on the role of oral microorganisms in diagnosing and treating oral diseases. While many studies have focused on unraveling microbial diversity, limited attention has been given to exploring the influence of community function, host genetic background, lifestyle factors, and biological events on the oral microbiota. The absence of such comprehensive data hampers a holistic understanding of the oral microbial community. Therefore, there is an urgent need for meaningful research in oral microbiology. Understanding the mechanisms that maintain the ecological balance of the oral microbiota will be instrumental in effectively reducing the incidence of oral diseases. To achieve this transition, we propose several testable hypotheses, methodological priorities, and translational objectives to guide future research.

### 9.1 Dental Caries

The age‐dependent microbial dynamics documented—with cariogenic taxa varying across children, adults, and the elderly—underscore the need for age‐stratified preventive strategies. Current evidence remains largely cross‐sectional; therefore, longitudinal studies are required to establish the temporal sequence of microbial succession and validate causality between specific microbial shifts and caries progression.

### 9.2 Periodontal and Peri‐Implant Diseases

The overlapping presence of keystone periodontopathogens in both conditions, alongside unique species such as *S. epidermidis* in peri‐implantitis, supports the ecological dysbiosis model. However, mechanistic insights remain scarce. Future research must pivot from compositional surveys to functional interaction networks and host–microbe crosstalk, paving the way for personalized interventions tailored to individual microbial and immunological profiles.

### 9.3 Salivary Gland Diseases

The multistage continuum from bacterial adhesion to biofilm formation and sialolith mineralization has been characterized, yet the dynamics of the salivary microbiome during active infection and in response to treatment remain largely uncharted. Longitudinal microbiome profiling of patients across the acute‐to‐chronic transition is needed to identify biomarkers of disease progression.

### 9.4 Oral Mucosal Diseases

Distinct microbial profiles across OLP subtypes, animal model evidence that microbial shifts precede clinical symptoms in leukoplakia, and RAS‐associated dysbiosis indices collectively highlight the complexity of host–microbe interactions. However, the causal direction between dysbiosis and epithelial damage remains unresolved. Prospective studies are required to elucidate causal relationships between microbial alterations and disease progression.

### 9.5 Oral Cancer

Five interconnected mechanistic pathways—chronic inflammation, cell cycle dysregulation, apoptosis inhibition, carcinogen production, and tissue invasion—have been documented, yet most evidence derives from in vitro or animal models. Emerging human studies linking microbial signatures to cancer risk and treatment outcomes underscore the need for multicenter prospective cohorts of patients with oral potentially malignant disorders and human‐relevant organoid models to validate causal relationships.

The oral microbiome field has matured from descriptive cataloging to a hypothesis‐driven discipline poised for clinical translation. By pursuing the specific hypotheses and objectives outlined above, future research can transform our understanding of oral diseases and deliver on the promise of microbiome‐based precision dentistry—where prevention, diagnosis, and therapy are tailored to each patient's unique microbial ecosystem.

## Author Contributions

**Zixi Kang:** writing – original draft, project administration. **Hong Huang:** writing – original draft, project administration. **Jin Lin:** software. **Yanan Niu:** software. **Jiaxin Chen:** software. **Zhengyuan Hu:** software. **Jiaxuan Tang:** software. **Peng Liu:** writing – review and editing, writing – original draft, supervision, resources. **Jun Qu:** writing – review and editing, writing – original draft, supervision, resources.

## Ethics Statement

The authors have nothing to report.

## Consent

The authors have nothing to report.

## Conflicts of Interest

None declared.

## Supporting information


**Table S1:** The possible association mechanism between oral microbiome and oral cancer.

## Data Availability

The authors have nothing to report.
