# Supplementary material for: Unraveling Oral Dysbiosis: Microbial Complexity in Common Oral Diseases
Source: Microbiologyopen. 2026 May 24;15(3):e70305. doi: 10.1002/mbo3.70305 (PMC13238755; doi:10.1002/mbo3.70305)
Supplement: Supplementary file 1 — Table S1: The possible association mechanism between oral microbiome and oral cancer. [file MBO3-15-e70305-s001.docx]

**Table S1: The possible association mechanism between oral microbiome and oral cancer.**

|  | Genus/Species | Possible Mechanisms of Association with Oral Cancer | Refs |
| --- | --- | --- | --- |
| Chronic Inflammation | *Aggregatibacter* | Production of proinflammatory cytokines | (1) |
|  | *Capnocytophaga* | Stimulation of inflammation | (2) |
|  | *Catonella* | Induction of chronic inflammation | (3) |
|  | *Eikenella corrodens* | Elevated production of IL-1, IL-6, IL-8, and TNF-α | (4) |
|  | *Enterococcus* | Maintaining chronic inflammation | (5) |
|  | *Fusobacterium nucleatum* | Secretion of IL-1β through activation of NLRP3 inflammasome | (6) |
|  | *Gemella* | IL-23 upregulation | (7) |
|  | *Mycoplasma salivarium* | Activation of NF-κB signal pathway | (8) |
|  | *Parvimonas* | Inflammation induction | (9) |
|  | *Porphyromonas gingivalis* | Chronic inflammation induction through IL-8, IL-6, TGF-β1, and TNF-α expression | (10) |
|  | *Prevotella intermedia* | IL-1, IL-6, IL-17, IL-23, and TNF-α expression | (11) |
|  | *Propionibacterium* | Production of IL-6 and IL-8 | (12) |
|  | *Pseudomonas aeruginosa* | Induction of inflammation through NF-κB pathway activation | (13) |
|  | *Streptococcus aureus* | Upregulation of COX-2 transcription; production of prostaglandins PGE2 | (14) |
|  | *Tannerella* | Proinflammatory cytokine production | (15) |
| Affecting the cell cycle/  Promoting cell proliferation | *Fusobacterium nucleatum* | Induction of epithelial–mesenchymal transition through lncRNA MIR4435-2HG/miR-296-5p/Akt2/SNAI1 pathway ;acceleration of cell cycle through p27 downregulation; activation of oncogenes cyclin D1 and myc through β-catenin pathway | (16)**;**(17) |
|  | *Mycoplasma salivarium* | P53 inhibition | (18) |
|  | *Porphyromonas gingivalis* | Stimulation of cell proliferation through upregulation of cyclins and p53 inhibition; induces noncanonical activation of beta-catenin and proteolytic dissociation of the beta-catenin destruction complex through the production of the gingipain family proteases, then contributes to the proliferative phenotype | (19–21) |
|  | *Prevotella intermedia* | Production of virulent factors (lipopolysaccharides, peptidoglycans, lipoteichoic acid | (22) |
|  | *Pseudomonas aeruginosa* | Secretion of LasI factor leading to downregulation of E-cadherin expression | (23) |
|  | *Streptococcus gordonii* | Suppression of epithelial–mesenchymal transition through decreasing ZEB2 expression | (24) |
|  | *Streptococcus aureus* | Induction of cyclin D1 overexpression | (25) |
| Inhibiting Apoptosis | *Porphyromonas gingivalis* | Induce inhibition of apoptosis through overstimulation of the JAK1/STAT3 signaling pathway; upregulation of miRNA-203 | (26)**;**(27) |
|  | *Acetobacter syzygii* | Possesses anticancer activity promoting the induction of apoptosis in oral cancer cells | (28) |
|  | *L. planarum* | Induce cancer cell apoptosis via upregulation of PTEN and downregulation of MAPK pathway | (25) |
|  | *Streptomyces* | Induction of cancer cell apoptosis | (29) |
| Production Of Carcinogens | *Aggregatibacter* | Production of hydrogen sulfide and methyl mercaptan inducing inflammation, cell proliferation, and tumor angiogenesis | (30) |
|  | *Enterococcus* | Increase in genomic instability linked to superoxide production | (31) |
|  | *Lactobacillus* | Some species produce lactate; L. fermentum produces hydrogen peroxide | (32) |
|  | *Porphyromonas gingivalis* | Production of reactive oxygen species, butyrate, and acetaldehyde | (33) |
|  | *Prevotella intermedia* | Production of hydrogen sulfide, methyl mercaptan, and acetaldehyde | (34) |
|  | *Pseudomonas aeruginosa* | Production of endotoxins such as LPS or flagella contribute to the induction of inflammation | (35) |
|  | *Rothia*  *Streptococcus anginosus*  *Streptococcus gordonii* *Streptococcus mitis*  *Streptococcus salivarius* | Acetaldehyde production | (36)**;**(37)**;**  (38) |
| Cellular Invasion | *Actinobacillus* | Upregulation of CCL20 in cancer cells | (39) |
|  | *Fusobacterium nucleatum* | P38 activation leading to increased production of MMP-13 and MMP-9 | (40) |
|  | *Prevotella intermedia* | Secretion of proteases | (41) |
|  | *Treponema denticola* | Dentilisin overexpression associated with increased tumor invasiveness | (42) |

References:

1. Mercer SD, Doherty C, Singh G, Willmott T, Cheesapcharoen T, Teanpaisan R, et al. Lactobacillus lysates protect oral epithelial cells from pathogen-associated damage, increase secretion of pro-inflammatory cytokines and enhance barrier integrity. Sci Rep. 2025 Feb 18;15(1):5894.

2. Wang SY, Cai Y, Hu X, Li F, Qian XH, Xia LY, et al. P. gingivalis in oral-prostate axis exacerbates benign prostatic hyperplasia via IL-6/IL-6R pathway. Mil Med Res. 2024 May 20;11(1):30.

3. Wei Y, Shi M, Nie Y, Wang C, Sun F, Jiang W, et al. Integrated analysis of the salivary microbiome and metabolome in chronic and aggressive periodontitis: a pilot study. Front Microbiol. 2022;13:959416.

4. Jud P, Wimmer G, Meinitzer A, Strohmaier H, Schwantzer G, Moazedi-Fürst F, et al. Periodontal disease and its association to endothelial dysfunction and clinical changes in limited systemic sclerosis: a case-control study. J Periodontal Res. 2023 Jun;58(3):621–33.

5. Quaglio AEV, Grillo TG, De Oliveira ECS, Di Stasi LC, Sassaki LY. Gut microbiota, inflammatory bowel disease and colorectal cancer. World J Gastroentero. 2022 Aug 14;28(30):4053–60.

6. Sun J, Feng S, Ding T, Wang T, Du L, Kang W, et al. Fusobacterium nucleatum dysregulates inflammatory cytokines and NLRP3 inflammasomes in oral cells. Oral Dis. 2024 Oct;30(7):4767–81.

7. Torres-Morales J, Mark Welch JL, Dewhirst FE, Borisy GG. Site-specialization of human oral gemella species. J Oral Microbiol. 2023;15(1):2225261.

8. Parfenyev SE, Vishnyakov IE, Efimova TN, Daks AA, Shuvalov OY, Fedorova OA, et al. Effect of infection by mycoplasma arginini and mycoplasma salivarium on the oncogenic properties of lung cancer cell line A549. Biochem Biophys Res Commun. 2024 Dec 3;736:150878.

9. Chen YY, Tan L, Su XL, Chen NX, Liu Q, Feng YZ, et al. NOD2 contributes to parvimonas micra-induced bone resorption in diabetic rats with experimental periodontitis. Mol Oral Microbiol. 2024 Dec;39(6):446–60.

10. Gasmi Benahmed A, Kumar Mujawdiya P, Noor S, Gasmi A. Porphyromonas gingivalis in the development of periodontitis: impact on dysbiosis and inflammation. Arch Razi Inst. 2022 Oct;77(5):1539–51.

11. Zhou Y, Qin Y, Ma J, Li Z, Heng W, Zhang L, et al. Heat-killed prevotella intermedia promotes the progression of oral squamous cell carcinoma by inhibiting the expression of tumor suppressors and affecting the tumor microenvironment. Exp Hematol Oncol. 2024 Mar 21;13(1):33.

12. Yu XQ, Mao JZ, Yang SY, Wang L, Yang CZ, Huang L, et al. Autocrine IL-8 contributes to propionibacterium acnes-induced proliferation and differentiation of HaCaT cells via AKT/FOXO1/ autophagy. Curr Med Sci. 2024 Oct;44(5):1058–65.

13. Jia X, Gu M, Dai J, Wang J, Zhang Y, Pang Z. Quercetin attenuates pseudomonas aeruginosa-induced acute lung inflammation by inhibiting PI3K/AKT/NF-κB signaling pathway. Inflammopharmacology. 2024 Apr;32(2):1059–76.

14. Butucel E, Balta I, Bundurus IA, Popescu CA, Iancu T, Venig A, et al. Natural antimicrobials promote the anti-oxidative inhibition of COX-2 mediated inflammatory response in primary oral cells infected with staphylococcus aureus, streptococcus pyogenes and enterococcus faecalis. Antioxid (Basel Switz). 2023 Apr 28;12(5):1017.

15. Loeurng V, Puth S, Hong SH, Lee YS, Radhakrishnan K, Koh JT, et al. A flagellin-adjuvanted trivalent mucosal vaccine targeting key periodontopathic bacteria. Nato Adv Sci Inst Se. 2024 Jul 8;12(7):754.

16. Zhang S, Li C, Liu J, Geng F, Shi X, Li Q, et al. Fusobacterium nucleatum promotes epithelial-mesenchymal transiton through regulation of the lncRNA MIR4435-2HG/miR-296-5p/Akt2/SNAI1 signaling pathway. FEBS J. 2020 Sep;287(18):4032–47.

17. Li Z, Liu Y, Huang X, Wang Q, Fu R, Wen X, et al. F. Nucleatum enhances oral squamous cell carcinoma proliferation via E-cadherin/β-catenin pathway. BMC oral health. 2024 May 2;24(1):518.

18. Parfenyev SE, Vishnyakov IE, Efimova TN, Daks AA, Shuvalov OY, Fedorova OA, et al. Effect of infection by mycoplasma arginini and mycoplasma salivarium on the oncogenic properties of lung cancer cell line A549. Biochem Biophys Res Commun. 2024 Dec 3;736:150878.

19. Shi J, Hao XY, Tong Y, Qian WB, Sun Y. SIRT6 alleviates senescence induced by porphyromonas gingivalis in human gingival fibroblasts. Mol Biol Rep. 2024 Sep 11;51(1):976.

20. Qiu Y, Tan X, Lei Z, Chen X, Chen J, Gong T, et al. A GntR family transcription factor in porphyromonas gingivalis regulates bacterial growth, acylpeptidyl oligopeptidase, and gingipains activity. Mol Oral Microbiol. 2023 Feb;38(1):48–57.

21. Fitzsimonds ZR, Liu C, Stocke KS, Yakoumatos L, Shumway B, Miller DP, et al. Regulation of olfactomedin 4 by porphyromonas gingivalis in a community context. ISME J. 2021 Sep;15(9):2627–42.

22. Peeran SW, Murugan M, Doggalli N, Fageeh H, Ibrahim W, Al-Ak’hali MS, et al. Herbal composite preparation and investigating its efficiency to inhibit biofilm formation and virulence factors of prevotella intermedia and porphyromonas gingivalis - formulation of mouthwash using a herbal composite and evaluating its anti-microbial activity. J Pharm Bioallied Sci. 2024 Apr;16(Suppl 2):S1574–84.

23. Fuentes-Zacarias P, Arzate-Castañeda DA, Sosa-González I, Villeda-Gabriel G, Morales-Méndez I, Osorio-Caballero M, et al. Pseudomonas aeruginosa induces spatio-temporal secretion of IL-1β, TNFα, proMMP-9, and reduction of epithelial E-cadherin in human alveolar epithelial type II (A549) cells. Acta Biochim Pol. 2021 May 4;68(2):207–15.

24. Ohshima J, Wang Q, Fitzsimonds ZR, Miller DP, Sztukowska MN, Jung YJ, et al. Streptococcus gordonii programs epithelial cells to resist ZEB2 induction by porphyromonas gingivalis. PNAS. 2019 Apr 23;116(17):8544–53.

25. Cao W, Liu Y, Zhang R, Zhang B, Wang T, Zhu X, et al. Homoharringtonine induces apoptosis and inhibits STAT3 via IL-6/JAK1/STAT3 signal pathway in Gefitinib-resistant lung cancer cells. Sci Rep. 2015 Jul 13;5:8477.

26. Bui FQ, Johnson L, Roberts J, Hung SC, Lee J, Atanasova KR, et al. Fusobacterium nucleatum infection of gingival epithelial cells leads to NLRP3 inflammasome-dependent secretion of IL-1β and the danger signals ASC and HMGB1. Cell Microbiol. 2016 Jul;18(7):970–81.

27. Yoshida Y. Analysis of the Butyrate-Producing Pathway in Porphyromonas gingivalis. Methods Mol Biol. 2021;2210:167–72.

28. Kumar SS. Can probiotics stop oral cancer progression? Evid-Based Dent. 2022 Mar;23(1):22–3.

29. Kouroshnia A, Zeinali S, Irani S, Sadeghi A. Induction of apoptosis and cell cycle arrest in colorectal cancer cells by novel anticancer metabolites of streptomyces sp. 801. Cancer Cell Int. 2022 Jul 26;22(1):235.

30. Ozuna H, Snider I, Belibasakis GN, Oscarsson J, Johansson A, Uriarte SM. Aggregatibacter actinomycetemcomitans and filifactor alocis: two exotoxin-producing oral pathogens. Front Oral Health. 2022;3:981343.

31. Tsukahara T, Makioka-Itaya Y, Takimoto H, Ijichi T. Oral supplementation of a cell preparation of enterococcus faecalis strain EC-12 stimulates superoxide dismutase production in the livers of healthy and arthritis-induced mice. J Clin Biochem Nutr. 2023 Jan;72(1):39–45.

32. Scillato M, Spitale A, Mongelli G, Privitera GF, Mangano K, Cianci A, et al. Antimicrobial properties of lactobacillus cell-free supernatants against multidrug-resistant urogenital pathogens. Microbiol Open. 2021 Feb;10(2):e1173.

33. Xu T, Dong Q, Luo Y, Liu Y, Gao L, Pan Y, et al. Porphyromonas gingivalis infection promotes mitochondrial dysfunction through Drp1-dependent mitochondrial fission in endothelial cells. Int J Oral Sci. 2021 Sep 3;13(1):28.

34. Lee YH, Kim H, Heo DW, Ahn IS, Auh QS. Volatile sulfide compounds and oral microorganisms on the inner surface of masks in individuals with halitosis during COVID-19 pandemic. Sci Rep. 2023 Feb 13;13(1):2487.

35. Qin S, Xiao W, Zhou C, Pu Q, Deng X, Lan L, et al. Pseudomonas aeruginosa: pathogenesis, virulence factors, antibiotic resistance, interaction with host, technology advances and emerging therapeutics. Signal Transduction Targeted Ther. 2022 Jun 25;7(1):199.

36. Xia M, Lei L, Zhao L, Xu W, Zhang H, Li M, et al. The dynamic oral-gastric microbial axis connects oral and gastric health: current evidence and disputes. npj Biofilms Microbiomes. 2025 Jan 2;11(1):1.

37. Fiore M, Minni A, Cavalcanti L, Raponi G, Puggioni G, Mattia A, et al. The impact of alcohol consumption and oral microbiota on upper aerodigestive tract carcinomas: a pilot study. Antioxid (Basel Switz). 2023 Jun 7;12(6):1233.

38. Galvin S, Honari B, Anishchuk S, Healy CM, Moran GP. Oral leukoplakia microbiome predicts the degree of dysplasia and is shaped by smoking and tooth loss. Oral Dis. 2025 Feb 4;

39. Abiko Y, Nishimura M, Kusano K, Nakashima K, Okumura K, Arakawa T, et al. Expression of MIP-3alpha/CCL20, a macrophage inflammatory protein in oral squamous cell carcinoma. Arch Oral Biol. 2003 Feb;48(2):171–5.

40. Suzuki R, Kamio N, Sugimoto K, Maruoka S, Gon Y, Kaneko T, et al. Periodontopathic bacterium fusobacterium nucleatum affects matrix metalloproteinase-9 expression in human alveolar epithelial cells and mouse lung. In Vivo (Athens Greece). 2022;36(2):649–56.

41. Nesbitt WE, Fukushima H, Leung KP, Clark WB. Coaggregation of prevotella intermedia with oral actinomyces species. Infect Immun. 1993 May;61(5):2011–4.

42. Goetting-Minesky MP, Godovikova V, Fenno JC. Approaches to understanding mechanisms of dentilisin protease complex expression in treponema denticola. Front Cell Infect Microbiol. 2021;11:668287.
